# Chronic In Vivo Interaction of Dendritic Cells Expressing the Ligand Rae-1ε with NK Cells Impacts NKG2D Expression and Function

**DOI:** 10.4049/immunohorizons.1700004

**Published:** 2017-05-01

**Authors:** Maelig G. Morvan, Marine Champsaur, Boris Reizis, Lewis L. Lanier

**Affiliations:** * Department of Microbiology and Immunology, University of California, San Francisco, San Francisco, CA 94143; † Parker Institute for Cancer Immunotherapy, University of California, San Francisco, San Francisco, CA 94143; ‡ Department of Pathology, New York University School of Medicine, New York, NY 10016; § Department of Microbiology and Immunology, Columbia University Medical Center, New York, NY 10032

## Abstract

To investigate how dendritic cells (DCs) interact with NK cells in vivo, we developed a novel mouse model in which Rae-1ε, a ligand of the NKG2D receptor, is expressed in cells with high levels of CD11c. In these CD11c-Rae1 mice, expression of Rae-1 was confirmed on all subsets of DCs and a small subset of B and T cells, but not on NK cells. DC numbers and activation status were unchanged, and NK cells in these CD11c-Rae1 mice presented the same Ly49 repertoire and maturation levels as their littermate wildtype controls. Early NK cell activation after mouse CMV infection was slightly lower than in wildtype mice, but NK cell expansion and viral control were comparable. Notably, we demonstrate that chronic interaction of NK cells with NKG2D ligand–expressing DCs leads to a reversible NKG2D down-modulation, as well as impaired NKG2D-dependent NK cell functions, including tumor rejection. In addition to generating a useful mouse model, our studies reveal in vivo the functional importance of the NK cell and DC cross-talk.

## INTRODUCTION

Natural killer group 2D (NKG2D) is an activating receptor expressed by all NK cells and subsets of αβ-TcR and γδ-TcR T cells. The ligands of NKG2D are frequently expressed by tumors of many cell types in humans and mice, by infected cells during viral infections, and by certain tissues in the context of autoimmune diseases (1, 2). Stimulatory signals delivered by NKG2D trigger cell-mediated cytotoxicity and cytokine secretion via the adapter protein DAP10 in humans (3) and by both DAP10 and DAP12 adapters in mice (4, 5). However, when NKG2D^+^ NK cells or T cells encounter their ligands, the receptor is downmodulated from the cell surface (6–9). The downmodulation acts as a feedback mechanism that prevents subsequent activation by target cells expressing NKG2D ligands (10). This process can be reversed after ligand removal (7).

By using a β-actin–*Raet1e* transgenic (RaeTg)mouse in which an NKG2D ligand is constitutively expressed on all cells and tissues, we have demonstrated that when NKG2D is chronically exposed to this ligand in vivo, its expression at the cell surface is downmodulated, and the NKG2D-dependent NK cell functions, including tumor elimination, are impaired (11). However, the ubiquitous and constitutive expression of retinoic acid early-inducible protein 1 ε (Rae-1ε) does not fully reflect the physiopathological situations in which NKG2D ligands are only expressed by limited cell subsets. Therefore, we developed a novel mouse model allowing us to specifically express Rae-1ε in any cell type or tissue. We focused our first application of this novel mouse model on dendritic cells (DCs) to determine whether DC-specific expression of the ligand would augment or suppress NK cell function upon interaction with DCs.

Cross-talk between NK cells and DCs is believed to play a major role during immune responses (12), and activated, but not resting, DCs have been shown to express NKG2D ligands (13–17). Several studies in mice and humans have reported NKG2D ligand expression on DCs stimulated with cytokines (18) or infected with pathogens (14). Whereas induction of NKG2D ligand expression on DCs has been described, there is little evidence of its effect on NK cell functions in vivo. This fact is particularly true for mouse models where the involvement of NKG2D in response to immune challenges is well described, but many of the cell types expressing its ligands in vivo are still to be identified (19). In the current study, we characterized how DC-specific expression of Rae-1ε impacts NK cell phenotype and function in vivo, particularly with respect to anti-tumor immunity.

## MATERIALS AND METHODS

### Mice

The Rosa26–*LoxP*-stop-*LoxP*–*Raet1e* mouse (R26-LSL-*Raet1e*) [Gt(ROSA)26Sor^tm1(Raet1e)Lll^] was generated by ligating the *Raet1e* cDNA into the pRosa26PAS plasmid (20), which was then line-arized and used for electroporation of C57BL/6 embryonic stem cells, followed by colony selection based on neomycin resistance. This mouse strain has been deposited in the Mouse Genome Informatics database (http://www.informatics.jax.org/) under accession number MGI:5823988. DNA was extracted from selected colonies, digested with Eco RV, and screened by genomic Southern blot hybridization using a 5′ probe to detect a 11 kb band for the wildtype allele, and a 3.8 kb band for the targeted allele, which includes an additional Eco RV site. R26-LSL-*Raet1e* mice were genotyped following the standard PCR protocol for *Rosa26* (21) and subsequent homozygous mice were bred to the *Itgax*cre (CD11c-Cre) transgenic mouse (22). Mice carrying the CD11c-Cre transgene were designated CD11c-Rae1 mice, whereas those lacking the CD11c-Cre transgene served as littermate controls. All mice were housed and used in compliance with the animal protocol approved by the University of California San Francisco Institutional Animal Care and Use Committee.

### Flow cytometry

Single-cell suspensions were obtained from various organs harvested after euthanasia or from peripheral blood after RBC lysis in ACK buffer. Cells were first incubated with an anti-CD16^+^ CD32 Ab (clone 2.4G2) at 10 μg/ml to block Fc receptors, and then stained with combinations of fluorescently labeled Abs specific to the following surface proteins: CD11b (clone M1/70), CD11c (clone N418), CD8α (clone 53-6.7), NK1.1 (clone PK136), TcRβ (clone H57-597), NKp46 (clone 29A1.4), NKG2D (clone CX5), CD27 (clone LG.3A10), CD69 (clone [^1^H].2F3), Ly49H (clone 3D10), and KLRG1 (clone 2F1). NK cell cytolytic activity was measured by assessing degranulation, or CD107a expression (clone 1D4B), in response to target cell stimulation (23). Production of IFN-γ was determined by intracellular staining (clone XMG1.2) after cell fixation and permeabilization (24). All previously listed Abs were acquired from the University of California San Francisco mAb Core, eBioscience, BioLegend, or TonBo Biosciences. Rae-1ε expression was determined with a pan-specific Rae-1 Ab (clone 186107; R&D Systems). Acquisition of the samples was performed on a LSRII flow cytometer (BD Biosciences) and the files generated were analyzed with FlowJo software (TreeStar).

### Mouse CMV infection

Mice were infected by i.p. injection of 5 × 10^4^ PFU of mouse CMV (MCMV, Smith strain) or a mutant form of MCMV lacking the viral proteinm 152(Δm152; generously provided by U. Koszinowski, Max von Pettenkofer-Institut, München, Germany) (25). Splenocytes were harvested after 1.5 d to assess early activation and IFN-γ production by NK cells, and after 7 and 14 d to determine expansion of MCMV-specific Ly49H^+^ NK cells.

### In vivo cytotoxicity assay

Mice were injected i.p. with a mix of the following cell lines: RMA, RMA/S (26), and RMA transduced to ectopically express Rae-1ε (27) at 1 × 10^6^ cells per mouse. A portion of the cell mix was kept for in vitro culture to assess cell line expansion in the absence of exposure to immune responses. After 2 d, mice were euthanized and the peritoneal cavity was rinsed with 10 ml of cold PBS. After RBCs were lysed with ACK buffer, the peritoneal cell content was stained with fluorescently labeled Abs for analysis by flow cytometry as described above, for the following markers: Ly49A (clone YE1) and H-2Kb (clone AF6-88.5), NKp46, and pan-specific Rae-1. RMA tumor cells stain brightly for Ly49A and H-2, whereas RMA/S stain brightly for Ly49A, but are H-2 negative; neither RMA nor RMA/S expresses NKp46.

### Splenocyte rejection assay

Splenocytes from C57BL/6 wildtype, β-2 microglobulin-deficient (28), and RaeTg (29) mice were mixed and labeled with CellTrace Violet (Life Technologies), and then injected intravenously at 4 × 10^6^ cells per mouse. One and two days following the cell transfer, recipient mice were bled and, after RBC lysis, peripheral blood leukocytes were stained for analysis by flow cytometry, using the markers listed above.

### Transplanted tumor growth

YAC-1 and A20 tumor lines transduced to stably express luciferase (30) were generously provided by Dr. T.V. Brennan (Duke University) and injected i.p. at 6 × 10^5^ cells per mouse. One and two days after transplantation, luciferin (Caliper Life Sciences) in sterile PBS was administered at 3 mg per mouse and the abdomens of anesthetized mice were imaged by an In Vivo Imaging System Spectrum (PerkinElmer) to quantify tumor growth.

## RESULTS

### Generation of the CD11c-Rae1 mouse model

**FIGURE 1:**
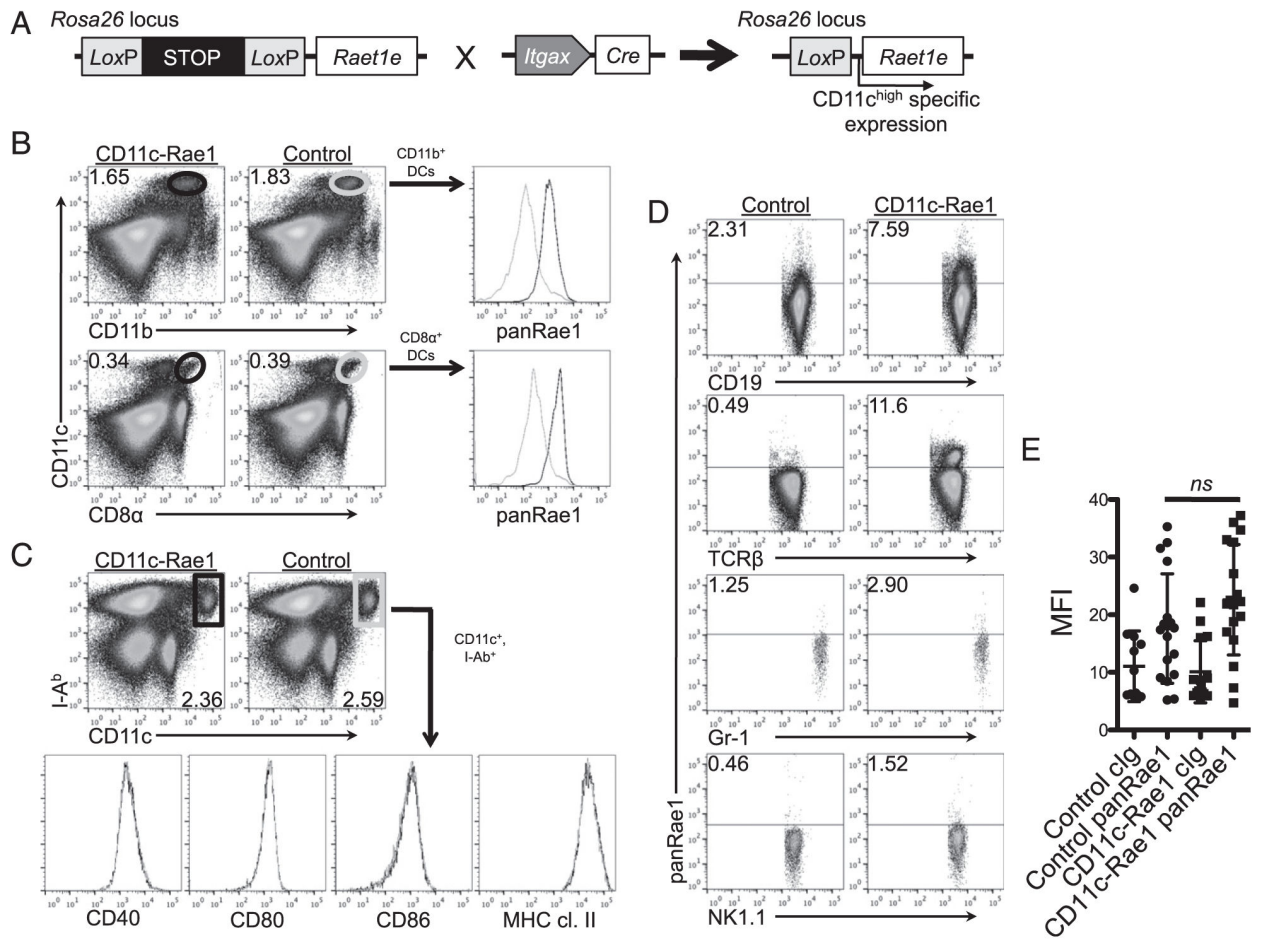
Generation of CD11c-Rae1 mice The construct inserted into the mouse *Rosa26* locus contains stop codons flanked by *LoxP* sites and followed by the *Raet1e* cDNA. When R26-LSL-*Raet1e* mice are crossed to transgenic *Itgax-*cre mice, CD11c^high^ cells preferentially express Rae-1ε (**A**). The CD11b^+^ (top panel) and CD8α^+^ (bottom panel) subsets of CD11c^high^ DCs from CD11c-Rae1 mice (black line) and littermate controls (gray line) were evaluated by flow cytometry for Rae-1 expression (**B**). DC maturation markers CD40, CD80, CD86, and MHC class II molecule expression levels were assessed in CD11c^high^ dendritic cells in CD11c-Rae1 mice (black line) and their littermate controls (gray line) (**C**). Expression of Rae-1 is determined on B cells (CD19^+^), T cells (TCRβ^+^), Gr-1^+^ myeloid cells, and NK cells (NK1.1^+^) (**D**). Data are representative of three experiments (*n* = 2–3 per group in each experiment). The median fluorescence intensity (MFI) for Rae-1 expression, and a control IgG (cIg), on NK cells (TCRβ^−^, NK1.1^+^ or NKp46^+^) is shown for CD11c-Rae1 mice (*n* = 18, ■) and their littermate controls (*n* = 17, ●) in three independent experiments (*p* = 0.1328) (**E**).

By inserting into the *Rosa26* locus a construct containing *LoxP* sites flanking stop codons, followed by the *Raet1e* cDNA, we created a knock-in mouse allowing for conditional expression of Rae-1ε (Fig. 1A). Mice homozygous for this R26-LSL-*Raet1e* allele were crossed to mice bearing a transgene in which the Cre recombinase is under the control of the *Itgax* (CD11c) promoter. In this latter CD11c-Cre transgenic mouse, CD11c^high^ cells, predominantly DCs, specifically express Cre (31). The resulting offspring were R26-LSL-*Raet1e*^+/−^, CD11c-Cre^+^ (CD11c-Rae1). Wildtype controls were R26-LSL-*Raet1e*^+/−^, Cre^−^ littermates.

### Characterization of CD11c-Rae1 DCs

The cell surface expression of Rae-1 was assessed by flow cytometry in the different DC subsets by using an anti-panspecific Rae-1 Ab (Fig. 1B). We confirmed that both CD11b^+^ and CD8α^+^ DCs expressed high levels of this NKG2D ligand. In addition, there was no difference in the frequency or number of the total DC population, characterized as CD11c^high^, MHC class II^+^, between the splenocytes of CD11c-Rae1 mice and their littermate controls (Fig. 1C). The expression levels of DC maturation markers, such as CD40, CD80, CD86, and MHC class II were also similar (Fig. 1C). Finally, cell surface expression of Rae-1 was detected on a small subset of B cells (CD19^+^) and T cells (TCRβ^+^), consistent with prior reports using this CD11c-Cre transgenic mouse (22) but not on Gr-1^+^ myeloid cells or NK cells themselves (Fig. 1D). Because this NKG2D ligand is not present at the surface of NK cells (Fig. 1E), this novel model was used to study how Rae-1ε expression on DCs impacts NK cell phenotype and function.

### NKG2D-independent cell phenotype and function in CD11c-Rae1 mice

**FIGURE 2:**
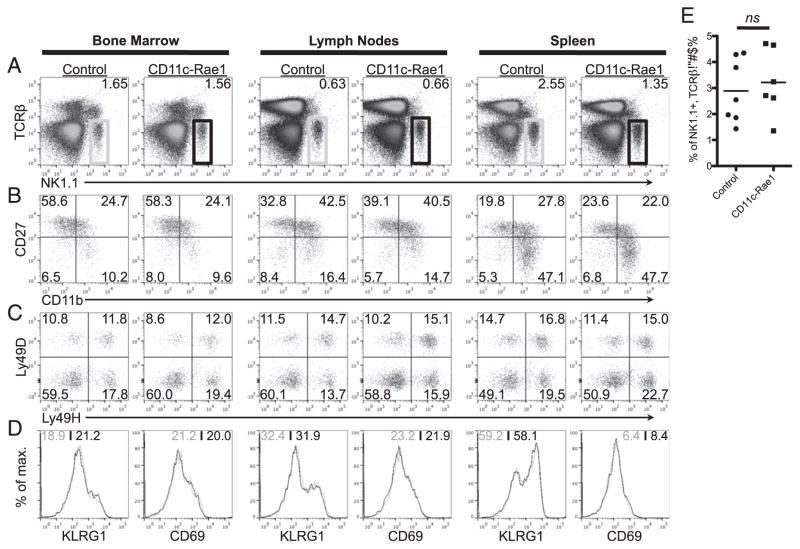
NK cells in CD11-Rae1 mice Leukocytes were isolated from the bone marrow, lymph nodes, and spleen from CD11c-Rae1 mice and their littermate controls to compare the frequency of NK1.1^+^, TCRβ^−^ NK cells (**A**), the expression of NK cell maturation markers CD11b and CD27 (**B**), the Ly49D and Ly49H repertoire (**C**), and the expression levels of two NK cell activation markers KLRG1 and CD69 (CD11c-Rae1, black line; control, black line) (**D**). Data are representative of three experiments (*n* = 2–3 per group in each experiment). The frequency of NK cells (TCRβ^−^, NK1.1^+^) is shown for CD11c-Rae1 mice (*n* = 6, ■) and their littermate controls (*n* = 7, ●) in three independent experiments (*p* = 0.65) (**E**).

We determined that the frequency and number of NK cells in the bone marrow, lymph nodes, and spleen were not different between CD11c-Rae1 mice and their littermate controls (Fig. 2A, 2E). NK cells of both genotypes express identical levels of CD11b and CD27 (Fig. 2B). This finding suggests that expression of the NKG2D ligand on DCs does not impact the development and maturation of NK cells in the CD11c-Rae1 mice. Furthermore, the Ly49 repertoire was similar in CD11c-Rae1 mice and their littermate controls, both for the frequency of NK cells expressing activating receptors (Fig. 2C) and inhibitory receptors (data not shown). We determined whether NK cells were more activated in the CD11c-Rae1 mice by evaluating the levels of KLRG1 and CD69, two activation markers on NK cells (32–34).

Unexpectedly, no difference was observed in the expression of either activation marker (Fig. 2D). This finding indicated that, in a normal physiological setting, NKG2D ligand expression on DCs does not activate NK cells.

**FIGURE 3:**
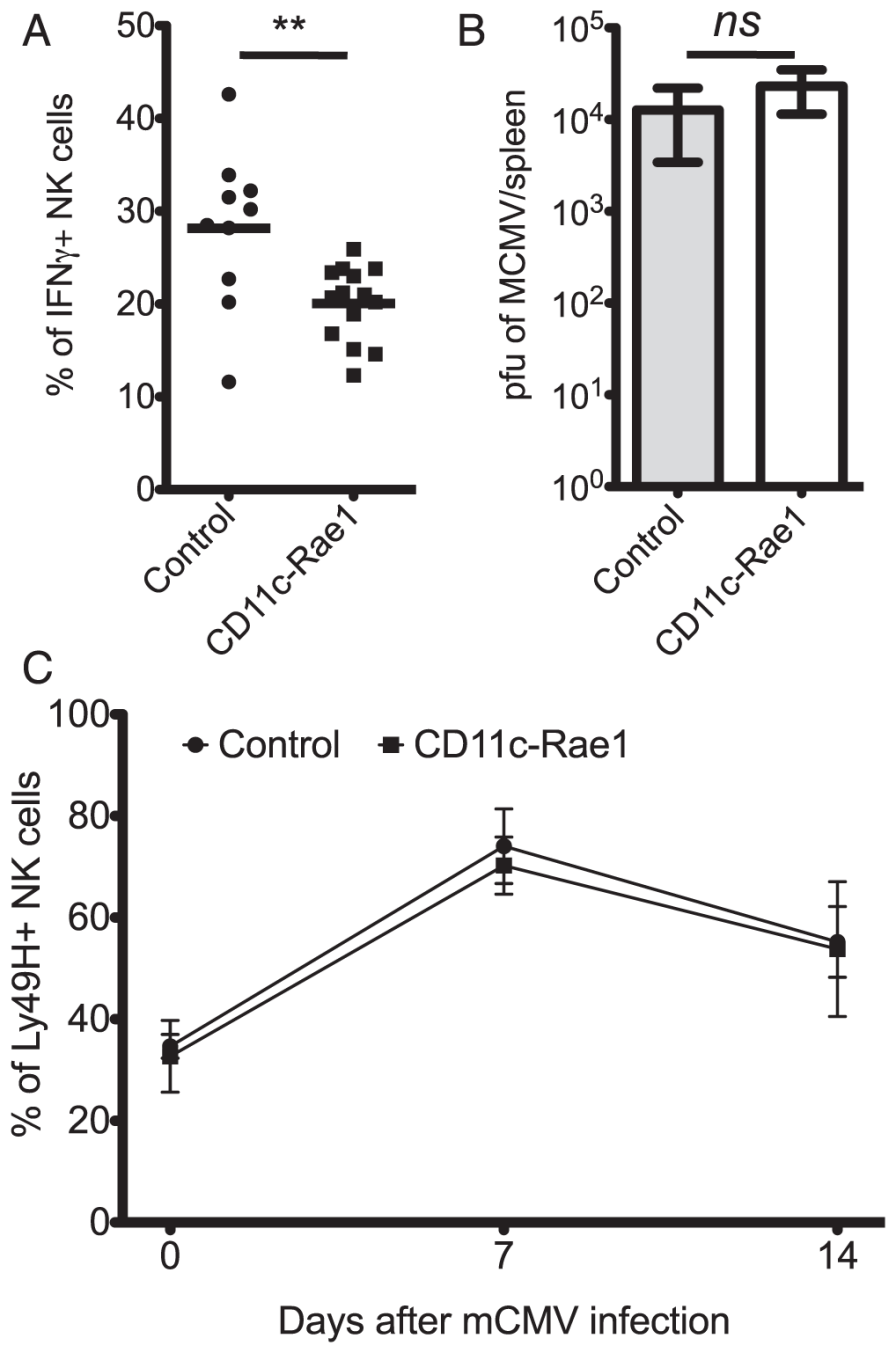
NK cells in CD11c-Rae1 mice after MCMV infection The frequency of splenic IFN-γ^+^ NK cells was lower (**p = 0.0047) 36 h after i.p. inoculation with MCMV for CD11c-Rae1 mice (*n* = 14, ■) than for their littermate controls (*n* = 10, ●) in five independent experiments (**A**). Viral burden in the spleen was measured on day 3 after i.p. inoculation with Δm152 MCMV in CD11c-Rae1 mice (*n* = 6, white bar) and their littermate controls (*n* = 6, gray bar) in two independent experiments (*p* = 0.12) (**B**). The frequency of splenic Ly49H^+^ NK cells was assessed 1 and 2 wk after i.p. inoculation with MCMV in CD11c-Rae1 mice (*n* = 19, ■) and their littermate controls (*n* = 12, ●) in five independent experiments (**C**).

To determine if NK cell activation would be affected in a pathological context, CD11c-Rae1 mice and their littermate controls were inoculated with MCMV, which leads to a systemic infection that is controlled by NK cells (35). Early NK cell activation, as assessed by CD69 and KLRG1 expression levels (data not shown) and intracellular IFN-γ production (Fig. 3A), was lower 36 h after MCMV infection in the CD11c-Rae1 mice, whereas the viral burden at 72 h remained unchanged (Fig. 3B). The frequency and number of Ly49H^+^ NK cells, which specifically expand in response to them 157 viral ligand (36), was also similar in CD11c-Rae1 and littermate control mice after 1 and 2 wk (Fig. 3C). This observation demonstrates that the NK cell response in the CD11c-Rae1 mouse is also not increased in the context of a systemic viral infection such as MCMV. Taken together, these results show that NKG2D-ligand expression on DCs does not substantially impact the NK cell phenotype and function that are independent of the NKG2D-mediated pathways during MCMV infection.

### Downmodulation of NKG2D expression

**FIGURE 4:**
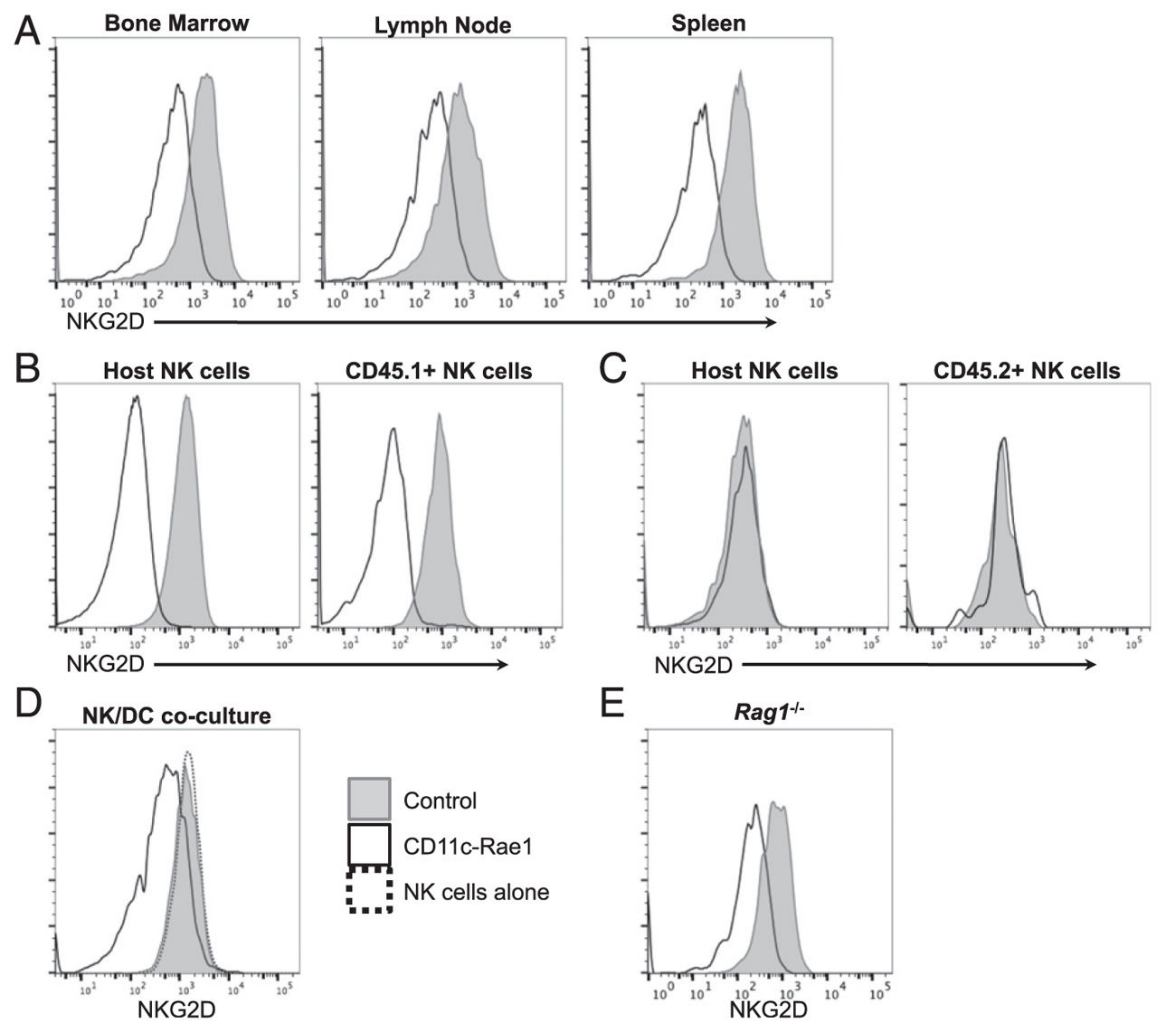
Expression of NKG2D on NK cells in CD11c-Rae1 mice Leukocytes were isolated from the bone marrow, lymph nodes, and spleen from CD11c-Rae1 mice (black line) and their littermate controls (gray line) to compare NKG2D expression levels on NK cells by flow cytometry. Data are representative of three experiments (*n* = 2–3 per group in each experiment) (**A**). Wildtype CD45.1^+^ splenocytes were adoptively transferred into CD11c-Rae1 mice (black line) and their littermate controls (gray line). After 2 d, NKG2D expression was assessed on both endogenous host NK cells (left panel) and transferred CD45.1^+^ NK cells (right panel). Data are representative of two experiments (*n* = 3 per group in each experiment) (**B**). Splenocytes from CD11c-Rae1 mice (black line) and their littermate controls (gray line) were adoptively transferred into wildtype CD45.1^+^ mice. After 2 d, NKG2D expression was determined on both endogenous host NK cells (left panel) and transferred CD45.2^+^ NK cells (right panel). Data are representative of two experiments (*n* = 3–4 per group in each experiment) (**C**). Splenic NK cells were isolated from wildtype mice and cocultured with sorted DCs from CD11c-Rae1 mice (black line), their littermate controls (gray line), or cultured alone (dotted line). After 1 d, NKG2D expression was assessed on NK cells by flow cytometry. Data are representative of two experiments (*n* = 3 per group in each experiment) (**D**). NKG2D expression was assessed by flow cytometry on peripheral blood NK cells (TCRβ^−^, NKp46^+^) from *Rag1*^−/−^ CD11c-Rae1 mice (black line) and their *Rag1*^−/−^ littermate controls (gray line). Data are representative of two experiments (*n* = 3 per group in each experiment) (**E**).

To determine how NKG2D ligand Rae-1ε expression on DCs affects the expression of the receptor on NK cells, we measured the levels of cell surface NKG2D by flow cytometry. NKG2D was strongly downmodulated from the NK cell surface, and this observation was made in all organs assessed (Fig. 4A). To assess if this downmodulation was caused by the interaction between NK cells and DCs in vivo, congenically marked wildtype NK cells from Ly5.1 mice were adoptively transferred into CD11c-Rae1 mice or their littermate controls. After 2 d, we confirmed that NKG2D expression was downmodulated on both the endogenous NK cells in the host and the transferred CD45.1^+^ NK cells when the recipients were CD11c-Rae1 mice (Fig. 4B). When their littermate controls were used as recipients, NKG2D expression remained at normal levels on both the host NK cells and the transferred CD45.1^+^ NK cells. In addition, we demonstrated that downmodulation of NKG2D expression on NK cells from CD11c-Rae1 mice could be recovered by performing the reverse transfer experiment (i.e., NK cells from CD11c-Rae1 mice transferred into wildtype recipients). In that case, NKG2D levels were identical after 2 d when congenically marked NK cells from both CD11c-Rae1 mice or their littermate controls were adoptively transferred into wildtype CD45.1 mice (Fig. 4C). In these transfer experiments, NKG2D downmodulation or recovery was observed as soon as after 1 d (data not shown). Finally, enriched NK cells from wildtype mice were cocultured in vitro with DCs isolated from CD11c-Rae1 mice or their littermate controls. Whereas NK cells cocultured overnight with CD11c-Rae1 DCs had decreased NKG2D levels, NK cells that were cocultured with control DCs had the same levels of NKG2D as NK cells that were left in culture alone (Fig. 4D). As Rae-1ε was also found on B and T cells, the CD11c-Rae1 mice were crossed to RAG-deficient mice, which have no B or T cells (37). In these *Rag1*^−^*^/^*^−^ CD11c-Rae1 mice, NKG2D was still downmodulated as the result of Rae-1ε being expressed only by DCs (Fig. 4E). As a whole, these data demonstrate that the interactions between NK cells and DCs expressing Rae-1ε result in a down-modulation of the NKG2D receptor from the NK cell surface that is quickly reversible.

### Impaired NKG2D-dependent NK cell function

**FIGURE 5:**
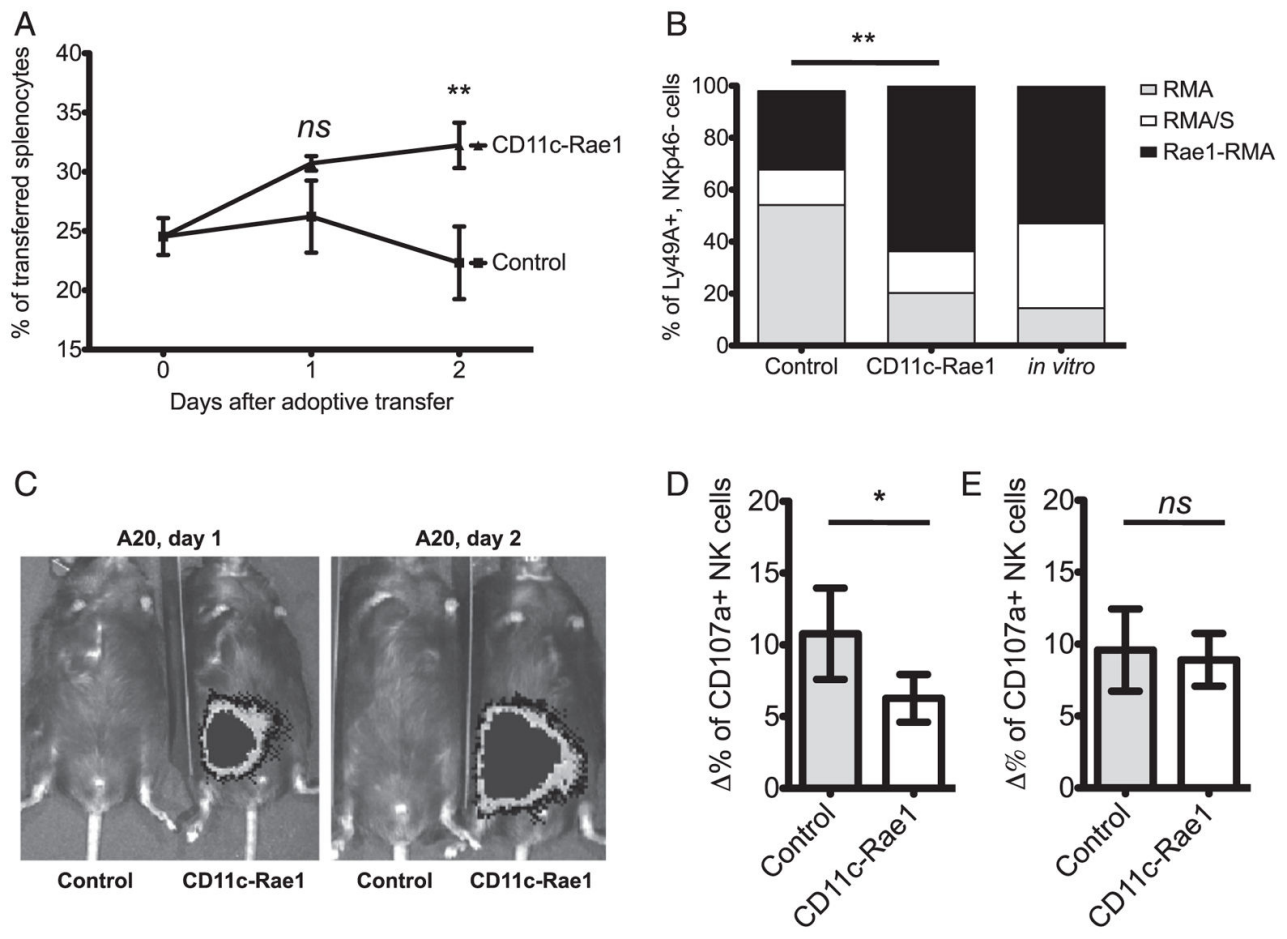
NKG2D-dependent NK cell function in CD11c-Rae1 mice A mixture of CellTrace Violet-labeled splenocytes from wildtype mice, *B2m*^−^*^/^*^−^ mice, and RaeTg mice was adoptively transferred to CD11c-Rae1 mice (*n* = 9, ▲) or their littermate controls (*n* = 6, ■) in two independent experiments; the frequency of RaeTg splenocytes was then determined by flow cytometry after 1 and 2 d, and was found to be significantly higher at day 2 (**p = 0.0082), but not at day 1 (*p* = 0.05) (**A**). A mixture of RMA (gray bars), RMA/S (white bars), and Rae1-RMA (black bars) cell lines was injected i.p. into CD11c-Rae1 mice (*n* = 4, middle) or their littermate controls (*n* = 8, left) or cultured in vitro (*n* = 6, right) in two independent experiments. After 2 d, the frequency of each cell line in the peritoneal cavity was determined by flow cytometry and the frequency of Rae1-RMA was found to be significantly higher (**p = 0.0087) in the CD11c-Rae1 mice (**B**). CD11c-Rae1 mice and their littermate controls were transplanted with luciferase-expressing A20 tumor cells; after 1 and 2 d, tumor burden was evaluated by imaging whole-body bioluminescence following luciferin administration. Data are representative of three experiments (*n* = 2 per group in each experiment) (**C**). Ex vivo degranulation (CD107a expression) after coculture with YAC-1 target cells was measured by flow cytometry for CD11c-Rae1 NK cells (white bar) and their littermate control NK cells (gray bar), in three independent experiments each performed in triplicate (*p = 0.02). Background (CD107a expression in the absence of target cells) was subtracted (**D**). Isolated NK cells were cultured overnight, and degranulation (CD107a expression) after coculture with YAC-1 target cells was measured by flow cytometry for CD11c-Rae1 NK cells (white bar) and their littermate control NK cells (gray bar), in three independent experiments each performed in triplicate (*p* = 0.59). Background (CD107a expression in the absence of target cells) was subtracted (**E**).

To establish whether the interaction of DCs with constitutive expression of an NKG2D ligand would increase or diminish NKG2D-dependent NK cell function, we injected intravenously a mix of fluorescently labeled splenocytes from wildtype, β2-microglobulin–deficient (38), and RaeTg mice. Splenocytes from *B2m*^−^*^/^*^−^ mice lack cell-surface MHC class I molecules and are susceptible to NK cell lysis, independently of NKG2D (39). In contrast, RaeTg splenocytes all express the NKG2D ligand and are lysed by NK cells using the NKG2D pathway (40). Two days after the transfer, the CD11c-Rae1 mice had significantly more RaeTg splenocytes than their littermate controls (*p* = 0.0082, Fig. 5A), whereas *B2m*^−^*^/^*^−^ splenocytes were eliminated efficiently (data not shown). This finding indicates that the in vivo NKG2D-dependent lysis of splenocytes constitutively expressing a NKG2D-ligand was impaired, but the NKG2D-independent rejection of MHC class I–deficient cells was not affected.

We also assessed if NKG2D-dependent cytotoxicity against tumor cell lines was affected. A mixture of three cell lines was injected i.p.: RMA, a MHC class I-bearing lymphoma that is resistant to NK cell cytotoxicity; RMA/S, a MHC class I-negative variant of RMA that is susceptible to NK cell-mediated cytotoxicity and rejection, independently of NKG2D; and Rae1-RMA, a RMA cell line transduced with *Raet1e*. This latter modification makes it susceptible to NKG2D-dependent NK cell cytotoxicity (27). Two days after the injection, the peritoneal cavity content was collected and assessed by flow cytometry. There were twice as many Rae1-RMA cells remaining in the CD11c-Rae1 mice, compared with their littermate controls (Fig. 5B). This in vivo cytotoxicity assay therefore confirms the observation that NK cell–mediated cytotoxicity triggered by NKG2D is substantially decreased in the CD11c-Rae1 mice.

We evaluated whether tumor rejection was also impaired in the CD11c-Rae1 mice. The CD11c-Rae1 and littermate control mice were inoculated with tumors endogenously expressing the NKG2D ligands, A20 and YAC-1 (41), which were transduced with luciferase. Bioluminescence after luciferin administration was used as a reporter for tumor burden. Whereas the littermate controls were able to reject the tumors as early as after 1 d (this very rapid rejection is consistent with NK cells, but not T cells, mediating the rejection), both the A20 (Fig. 5C) and YAC-1 (data not shown) tumors were able to be established and grow in the CD11c-Rae1 mice. In addition, NK cells isolated from CD11c-Rae1 mice exhibited significantly lower degranulation ex vivo against YAC-1 target cells compared with NK cells isolated from their littermate controls (Fig. 5D), which corroborates our observations made in vivo. Finally, after in vitro culture overnight, degranulation against YAC-1 target cells was comparable between NK cells isolated from CD11c-Rae1 mice and NK cells from their littermate controls (Fig. 5E), showing that these NK cells can recover their NKG2D-dependent function and there is no intrinsic defect in NKG2D signaling. These results notably demonstrate that although NKG2D-independent NK cell function is intact in the CD11c-Rae1 mice, NK cell–mediated lysis and tumor rejection using three different NKG2D ligand–bearing tumor cell lines are dramatically diminished when DCs constitutively express the NKG2D ligand Rae-1ε, but this functional impairment is reversible.

## DISCUSSION

Interaction of NKG2D with its ligands results in NK cell activation and downmodulation of the receptor from the NK cell surface. Studies suggest this interaction also mediates the cross-talk between NK cells and DCs. We developed a novel mouse model to study the functional impact on immune cells resulting from the specific expression of the NKG2D ligand Rae-1ε in any cell type or tissue. Our findings, using this mouse model, represent an advancement from previous results with transgenic mice that only had ubiquitous expression of NKG2D ligands (11), including on NK cells themselves, or expression restricted to epithelial tissues (8). For these studies, we first used the CD11c-Cre transgenic mouse to selectively express Rae-1ε in DCs.

With this model, we generated CD11c-Rae1 mice in which Rae-1ε expression was preferentially expressed in DC subsets, so we could assess the potential role of the NKG2D pathway in the cross-talk between NK cells and DCs. Despite the NKG2D ligand being expressed on such a small subset of cells, strikingly we observed a strong downmodulation of NKG2D expression from the NK cell surface in all NK cells in all tissues in these mice. This downmodulation happens very quickly as NKG2D expression on NK cells transferred into CD11c-Rae1 mice was already lower after 1 d. It was also reversible as NK cells from CD11c-Rae1 mice rapidly recovered NKG2D to normal levels when they were adoptively transferred into wildtype mice. Although Rae-1ε was also expressed on a very small subset of Band T cells, we confirmed that the downmodulation of NKG2D can result from the interaction with DCs, but not other Rae-1ε–expressing lymphocytes, by crossing CD11c-Rae1 mice to mice lacking B and T cells. These results strongly demonstrate that all the NK cells are interacting with DCs in vivo.

As with our previous studies of RaeTg mice in which the NKG2D ligand is ubiquitously expressed (11), we now show that binding of NKG2D to its ligand in the CD11c-Rae1 mouse does not affect NK cell development or their Ly49 receptor repertoire. Moreover, it does not impair general NK cell function. However, restricting Rae-1ε expression to DCs is still sufficient to downmodulate NKG2D expression and substantially impair NKG2D mediated NK cell function as measured by cytotoxicity and tumor rejection. The presence of this activating ligand on DCs did not increase NK cell activation or the anti-viral response against MCMV infection. Although this finding could suggest that the NKG2D pathway plays a dispensable role for NK cell activation by DCs, in contrast to prior studies suggesting a role for DC–NK cross-talk (42), the naturally increased NKG2D ligand expression occurring in wildtype mice could be sufficient to generate optimal NK cell activation, with the additional ligand in the CD11c-Rae1 mice not amplifying this signal for NK cells. However, as activated CD8^+^ T cells also express NKG2D in mice (43), it will be of interest to determine if increased NKG2D ligand on APCs might result in enhanced CD8^+^ T cell responses and if NKG2D has an important costimulatory role in this context. In the context of MCMV infection, the amplitude of the CD8^+^ T cell response in CD11c-Rae1 mice does not seem to be affected (data not shown).

Transgenic mice with Cre expression driven by many other cell-specific promoters are readily available. For example, this mouse model can be used to address whether expression of NKG2D ligands in other myeloid cells, including macrophages or neutrophils, would lead to similar observations to those we made here. Also, NK cell interactions with other lymphocytes such as B and T cells, as well as between NK cells themselves, have been reported to be mediated by NKG2D (44–47). Therefore, exclusive or inducible expression of NKG2D ligands in these specific cell subsets might help our understanding of the mechanisms involved in these NK cell interactions. Notably, our conditional knock-in mouse model represents a valuable tool to address how the restricted expression of Rae-1ε in some tissues could influence the outcome of some pathologies in which NKG2D has been suggested to have an impact. It is now possible to use transgenic mice for which the Cre recombinase is expressed under the control of the same promoter as an oncogene. Thus, our R26-LSL-*Raet1e* mouse could help understand how the expression of an NKG2D ligand influences primary tumorigenesis and tumor surveillance by the immune system. Also, as the NKG2D pathway has been described to play a potential role in certain autoimmune diseases such as diabetes, rheumatoid arthritis, multiple sclerosis, Crohn’s disease, lupus, or celiac disease (48), our new mouse model provides the ability to selectively express the NKG2D ligand in the targeted tissue with the corresponding Cre transgenic mouse. In addition, this mouse model can be used to study the cell subsets involved in NKG2D-dependent bone marrow rejection (40) or the impact of Rae-1ε expression on decidual NK cell function (49).

Recently we reported that NKG2D ligands are preferentially expressed on myeloid cells in glioblastoma patients, both myeloid cells within the tumor as well as circulating monocytes (50). Further, we observed that the NK cells and CD8^+^ T cells in these patients demonstrate lower levels of NKG2D and have impaired NK cell–mediated cytolytic function. After removal from the cancer patient and culture in vitro, NKG2D levels were restored on NK cells. Our CD11c-Rae1 mouse can be used as a model to mimic this phenomenon that occurs in cancer patients to design therapies that may restore NKG2D expression and function in vivo. Studies using CD11c-Rae1 mice with established transplantable solid tumors or in primary tumorigenesis models may provide valuable insight on the importance of NKG2D ligand expression by third-party cells on tumor surveillance and elimination.

Promising treatments of cancers and certain autoimmune or infectious diseases might be based on selectively manipulating NKG2D or NKG2D-ligand expression. Some tumor escape variants (51) or cells infected by some pathogens (52) do not express NKG2D ligands and could be treated by inducing those ligands on the cells (53). Yet, patients with consistently high levels of NKG2D ligands and an impaired NKG2D pathway in immune cells might benefit from therapeutic approaches aimed at reducing ligand expression on tumor cells and on other nontransformed cells, or at recovering normal levels of NKG2D expression on immune cells.

## Abbreviations used in this article

DC: dendritic cell
MCMV: mouse CMV
Rae-1: retinoic acid early-inducible protein 1
RaeTg: β-actin–*Raet1e* transgenic
R26-LSL-*Raet1e*: Rosa26–*LoxP*-stop-*LoxP*–*Raet1e*

## References

[1] Raulet DH, Gasser S, Gowen BG, Deng W, Jung H (2013). Regulation of ligands for the NKG2D activating receptor. Annu Rev Immunol.

[2] Lanier LL (2015). NKG2D receptor and its ligands in host defense. Cancer Immunol Res.

[3] Wu J, Song Y, Bakker ABH, Bauer S, Spies T, Lanier LL, Phillips JH (1999). An activating immunoreceptor complex formed by NKG2D and DAP10. Science.

[4] Diefenbach A, Tomasello E, Lucas M, Jamieson AM, Hsia JK, Vivier E, Raulet DH (2002). Selective associations with signaling proteins determine stimulatory versus costimulatory activity of NKG2D. Nat Immunol.

[5] Gilfillan S, Ho EL, Cella M, Yokoyama WM, Colonna M (2002). NKG2D recruits two distinct adapters to trigger NK cell activation and costimulation. Nat Immunol.

[6] Coudert JD, Zimmer J, Tomasello E, Cebecauer M, Colonna M, Vivier E, Held W (2005). Altered NKG2D function in NK cells induced by chronic exposure to NKG2D ligand-expressing tumor cells. Blood.

[7] Ogasawara K, Hamerman JA, Hsin H, Chikuma S, Bour-Jordan H, Chen T, Pertel T, Carnaud C, Bluestone JA, Lanier LL (2003). Impairment of NK cell function by NKG2D modulation in NOD mice. Immunity.

[8] Oppenheim DE, Roberts SJ, Clarke SL, Filler R, Lewis JM, Tigelaar RE, Girardi M, Hayday AC (2005). Sustained localized expression of ligand for the activating NKG2D receptor impairs natural cytotoxicity in vivo and reduces tumor immunosurveillance. Nat Immunol.

[9] Wiemann K, Mittrücker HW, Feger U, Welte SA, Yokoyama WM, Spies T, Rammensee HG, Steinle A (2005). Systemic NKG2D down-regulation impairs NK and CD8 T cell responses in vivo. J Immunol.

[10] Molfetta R, Quatrini L, Zitti B, Capuano C, Galandrini R, Santoni A, Paolini R (2016). Regulation of NKG2D expression and signaling by endocytosis. Trends Immunol.

[11] Champsaur M, Beilke JN, Ogasawara K, Koszinowski UH, Jonjic S, Lanier LL (2010). Intact NKG2D-independent function of NK cells chronically stimulated with the NKG2D ligand Rae-1. J Immunol.

[12] Thomas R, Yang X (2016). NK-DC crosstalk in immunity to microbial infection. J Immunol Res.

[13] Andoniou CE, van Dommelen SLH, Voigt V, Andrews DM, Brizard G, Asselin-Paturel C, Delale T, Stacey KJ, Trinchieri G, Degli-Esposti MA (2005). Interaction between conventional dendritic cells and natural killer cells is integral to the activation of effective antiviral immunity. Nat Immunol.

[14] Draghi M, Pashine A, Sanjanwala B, Gendzekhadze K, Cantoni C, Cosman D, Moretta A, Valiante NM, Parham P (2007). NKp46 and NKG2D recognition of infected dendritic cells is necessary for NK cell activation in the human response to influenza infection. J Immunol.

[15] Ebihara T, Masuda H, Akazawa T, Shingai M, Kikuta H, Ariga T, Matsumoto M, Seya T (2007). Induction of NKG2D ligands on human dendritic cells by TLR ligand stimulation and RNA virus infection. Int Immunol.

[16] Jinushi M, Takehara T, Tatsumi T, Kanto T, Groh V, Spies T, Suzuki T, Miyagi T, Hayashi N (2003). Autocrine/paracrine IL-15 that is required for type I IFN-mediated dendritic cell expression of MHC class I-related chain A and B is impaired in hepatitis C virus infection. J Immunol.

[17] Schrama D, Terheyden P, Otto K, Kämmerer U, Bröcker EB, Lühder F, Cosman D, Andersen MH, Becker JC (2006). Expression of the NKG2D ligand UL16 binding protein-1 (ULBP-1) on dendritic cells. Eur J Immunol.

[18] Jinushi M, Takehara T, Kanto T, Tatsumi T, Groh V, Spies T, Miyagi T, Suzuki T, Sasaki Y, Hayashi N (2003). Critical role of MHC class I-related chain A and B expression on IFN-α-stimulated dendritic cells in NK cell activation: impairment in chronic hepatitis C virus infection. J Immunol.

[19] Eagle RA, Jafferji I, Barrow AD (2009). Beyond stressed self: evidence for NKG2D ligand expression on healthy cells. Curr Immunol Rev.

[20] Srinivas S, Watanabe T, Lin CS, William CM, Tanabe Y, Jessell TM, Costantini F (2001). Cre reporter strains produced by targeted insertion of EYFP and ECFP into the ROSA26 locus. BMC Dev Biol.

[21] Soriano P (1999). Generalized lacZ expression with the ROSA26 Cre reporter strain. Nat Genet.

[22] Caton ML, Smith-Raska MR, Reizis B (2007). Notch-RBP-J signaling controls the homeostasis of CD8-dendritic cells in the spleen. J Exp Med.

[23] Alter G, Malenfant JM, Altfeld M (2004). CD107a as a functional marker for the identification of natural killer cell activity. J Immunol Methods.

[24] Hussell T, Openshaw PJ (1998). Intracellular IFN-gamma expression in natural killer cells precedes lung CD8+ T cell recruitment during respiratory syncytial virus infection. J Gen Virol.

[25] Bubić I, Wagner M, Krmpotić A, Saulig T, Kim S, Yokoyama WM, Jonjić S, Koszinowski UH (2004). Gain of virulence caused by loss of a gene in murine cytomegalovirus. J Virol.

[26] Ljunggren HG, Ohlén C, Höglund P, Franksson L, Kärre K (1991). The RMA-S lymphoma mutant; consequences of a peptide loading defect on immunological recognition and graft rejection. Int J Cancer Suppl.

[27] Cerwenka A, Baron JL, Lanier LL (2001). Ectopic expression of retinoic acid early inducible-1 gene (RAE-1) permits natural killer cell-mediated rejection of a MHC class I-bearing tumor in vivo. Proc Natl Acad Sci USA.

[28] Koller BH, Marrack P, Kappler JW, Smithies O (1990). Normal development of mice deficient in beta 2M, MHC class I proteins, and CD8+ T cells. Science.

[29] Ehrlich LIR, Ogasawara K, Hamerman JA, Takaki R, Zingoni A, Allison JP, Lanier LL (2005). Engagement of NKG2D by cognate ligand or antibody alone is insufficient to mediate costimulation of human and mouse CD8+ T cells. J Immunol.

[30] Brennan TV, Lin L, Brandstadter JD, Rendell VR, Dredge K, Huang X, Yang Y (2016). Heparan sulfate mimetic PG545-mediated antilymphoma effects require TLR9-dependent NK cell activation. J Clin Invest.

[31] Abram CL, Roberge GL, Hu Y, Lowell CA (2014). Comparative analysis of the efficiency and specificity of myeloid-Cre deleting strains using ROSA-EYFP reporter mice. J Immunol Methods.

[32] Fogel LA, Sun MM, Geurs TL, Carayannopoulos LN, French AR (2013). Markers of nonselective and specific NK cell activation. J Immunol.

[33] Huntington ND, Tabarias H, Fairfax K, Brady J, Hayakawa Y, Degli-Esposti MA, Smyth MJ, Tarlinton DM, Nutt SL (2007). NK cell maturation and peripheral homeostasis is associated with KLRG1 up-regulation. J Immunol.

[34] Kamimura Y, Lanier LL (2015). Homeostatic control of memory cell progenitors in the natural killer cell lineage. Cell Rep.

[35] Scalzo AA, Fitzgerald NA, Wallace CR, Gibbons AE, Smart YC, Burton RC, Shellam GR (1992). The effect of the Cmv-1 resistance gene, which is linked to the natural killer cell gene complex, is mediated by natural killer cells. J Immunol.

[36] Dokun AO, Kim S, Smith HRC, Kang HSP, Chu DT, Yokoyama WM (2001). Specific and nonspecific NK cell activation during virus infection. Nat Immunol.

[37] Mombaerts P, Iacomini J, Johnson RS, Herrup K, Tonegawa S, Papaioannou VE (1992). RAG-1-deficient mice have no mature B and T lymphocytes. Cell.

[38] Höglund P, Ohlén C, Carbone E, Franksson L, Ljunggren HG, Latour A, Koller B, Kärre K (1991). Recognition of beta 2-microglobulin-negative (beta 2m-) T-cell blasts by natural killer cells from normal but not from beta 2m-mice: nonresponsiveness controlled by beta 2m-bone marrow in chimeric mice. Proc Natl Acad Sci USA.

[39] Raulet DH, Vance RE (2006). Self-tolerance of natural killer cells. Nat Rev Immunol.

[40] Ogasawara K, Benjamin J, Takaki R, Phillips JH, Lanier LL (2005). Function of NKG2D in natural killer cell-mediated rejection of mouse bone marrow grafts. Nat Immunol.

[41] Diefenbach A, Jamieson AM, Liu SD, Shastri N, Raulet DH (2000). Ligands for the murine NKG2D receptor: expression by tumor cells and activation of NK cells and macrophages. Nat Immunol.

[42] Zitvogel L, Terme M, Borg C, Trinchieri G, Compans RW, Cooper MD, Honjo T, oprowski H, Melchers F, Oldstone MBA, Olsnes S, Potter M, Vogt PK, Wagner H (2006). Dendritic cell-NK cell cross-talk: regulation and physiopathology. Immunobiology of Natural Killer Cell Receptors. Current Topics in Microbiology and Immunology.

[43] Jamieson AM, Diefenbach A, McMahon CW, Xiong N, Carlyle JR, Raulet DH (2002). The role of the NKG2D immunoreceptor in immune cell activation and natural killing. Immunity.

[44] Cerboni C, Zingoni A, Cippitelli M, Piccoli M, Frati L, Santoni A (2007). Antigen-activated human T lymphocytes express cellsurface NKG2D ligands via an ATM/ATR-dependent mechanism and become susceptible to autologous NK-cell lysis. Blood.

[45] Nakamura K, Nakayama M, Kawano M, Amagai R, Ishii T, Harigae H, Ogasawara K (2013). Fratricide of natural killer cells dressed with tumor-derived NKG2D ligand. Proc Natl Acad Sci USA.

[46] Nowbakht P, Ionescu MCS, Rohner A, Kalberer CP, Rossy E, Mori L, Cosman D, De Libero G, Wodnar-Filipowicz A (2005). Ligands for natural killer cell-activating receptors are expressed upon the maturation of normal myelomonocytic cells but at low levels in acute myeloid leukemias. Blood.

[47] Rabinovich BA, Li J, Shannon J, Hurren R, Chalupny J, Cosman D, Miller RG (2003). Activated, but not resting, T cells can be recognized and killed by syngeneic NK cells. J Immunol.

[48] Van Belle TL, von Herrath MG (2009). The role of the activating receptor NKG2D in autoimmunity. Mol Immunol.

[49] Marlin R, Duriez M, Berkane N, de Truchis C, Madec Y, Rey-Cuille MA, Cummings JS, Cannou C, Quillay H, Barré-Sinoussi F (2012). Dynamic shift from CD85j/ILT-2 to NKG2D NK receptor expression pattern on human decidual NK during the first trimester of pregnancy. PLoS One.

[50] Crane CA, Austgen K, Haberthur K, Hofmann C, Moyes KW, Avanesyan L, Fong L, Campbell MJ, Cooper S, Oakes SA (2014). Immune evasion mediated by tumor-derived lactate dehydrogenase induction of NKG2D ligands on myeloid cells in glioblastoma patients. Proc Natl Acad Sci USA.

[51] Belting L, Hömberg N, Przewoznik M, Brenner C, Riedel T, Flatley A, Polić B, Busch DH, Röcken M, Mocikat R (2015). Critical role of the NKG2D receptor for NK cell-mediated control and immune escape of B-cell lymphoma. Eur J Immunol.

[52] Jonjić S, Polić B, Krmpotić A (2008). Viral inhibitors of NKG2D ligands: friends or foes of immune surveillance?. Eur J Immunol.

[53] Spear P, Wu MR, Sentman ML, Sentman CL (2013). NKG2D ligands as therapeutic targets. Cancer Immun.

